# Characterization of the Jumbo Squid (*Dosidicus gigas*) Skin By-Product by Shotgun Proteomics and Protein-Based Bioinformatics

**DOI:** 10.3390/md18010031

**Published:** 2019-12-29

**Authors:** Mónica Carrera, Josafat Marina Ezquerra-Brauer, Santiago P. Aubourg

**Affiliations:** 1 Department of Food Technology, Marine Research Institute (IIM), Spanish National Research Council (CSIC), 36208 Vigo, Pontevedra, Spain; saubourg@iim.csic.es; 2 Department of Food Research and Postgraduate, University of Sonora, 83100 Hermosillo, Sonora, Mexico; ezquerra@guayacan.uson.mx

**Keywords:** *Dosidicus gigas*, squid, skin, by-product, shotgun proteomics, mass spectrometry, protein-based bioinformatics, bioactive peptides

## Abstract

Jumbo squid (*Dosidicus gigas*) is one of the largest cephalopods, and represents an important economic fishery in several regions of the Pacific Ocean, from southern California in the United States to southern Chile. Large and considerable discards of this species, such as skin, have been reported to constitute an important source of potential by-products. In this paper, a shotgun proteomics approach was applied for the first time to the characterization of the jumbo squid (*Dosidicus gigas*) skin proteome. A total of 1004 different peptides belonging to 219 different proteins were identified. The final proteome compilation was investigated by integrated *in-silico* studies, including gene ontology (GO) term enrichment, pathways, and networks studies. Potential new valuable bioactive peptides such as antimicrobial, bioactive collagen peptides, antihypertensive and antitumoral peptides were predicted to be present in the jumbo squid skin proteome. The integration of the global proteomics results and the bioinformatics analysis of the jumbo squid skin proteome show a comprehensive knowledge of this fishery discard and provide potential bioactive peptides of this marine by-product.

## 1. Introduction

Marine by-products are the body parts of marine species that are removed before they reach the final consumer in order to improve their preservation, reduce the shipping weight, and increase the quality of the main product [1,2]. These organic materials are the main concern for current fishery management policies and legislation because they represent a significant source of valuable compounds such as proteins, minerals and lipids. In fact, from 2019 new regulations of fishery landing in the European Commission (EU) (European Commission Regulation (EU) No 1380/2013) oblige to keep and not discard all the species that are caught that are subjected to quota as well as underutilized commercial species [3]. For this reason, valorization solutions of marine discards biomasses have to be implemented. These new potential bioactive compounds could be used for human nutrition, as well as for their functional properties for nutraceutical, pharmaceutical, and cosmeceuticals industries [4,5,6,7]. 

Jumbo squid (*Dosidicus gigas*), also known as Humboldt squid, is one of the largest cephalopods and lives in the waters of the Humboldt Current in the eastern Pacific Ocean. It represents an important economic fishery resource in a wide number of countries such as Chile, Peru, Japan, and Mexico [8]. Nevertheless, only the jumbo squid mantle is marketed. During its processing, large amounts (up to 60% of whole weight) of squid off-products, such as skin, heads, fins, tentacles, and guts are generated and discarded [9].

By-products of the jumbo squid have recently attracted great attention due to the discovery of the presence of several relevant bioactive compounds. These include valuable and profitable bio-ingredients such as chitin, chitosan, collagen, gelatin, and pigments [10,11,12,13,14].

Particularly, the skin constitutes a significant sub-product in the jumbo squid fishery industry. Skin is actually a biological cooperative tissue formed by four different tissue types (epithelial, connective, muscle, and nerve tissues). Peptides derived from a tryptic hydrolysate of jumbo squid skin exhibited strong inhibition of lipid peroxidation that was much higher than the natural antioxidant α-tocopherol [15]. Skin molecules as xanthommatin also showed in vitro antioxidant effects [16]. Additionally, cytotoxic, antimicrobial, anti-biofilm, angiotensin converting enzyme (ACE)-inhibitory peptides, and anti-tumoral properties have been demonstrated for skin ink and the hydrolyzed skin of different squid species [14,17,18]. Recently, the inclusion on ice of a jumbo squid skin extract led to a remarkable microbial inhibition and a significant shelf life extension during fish chilled storage [19,20]. However, the global characterization of proteins and peptides from jumbo skin proteome has not been investigated to date.

Proteomics, as the discipline for the large-scale analysis of proteins of a particular biological system, has greatly contributed to the assessment of quality, safety, and bioactivity of seafood products [21,22,23,24]. In a shotgun proteomics approach, a mixture of proteins is digested with a protease (i.e., trypsin), and the resulting mixture of peptides is then analyzed by liquid chromatography coupled to tandem mass spectrometry (LC-MS/MS) [25]. Using database searching programs, like SEQUEST [26] or Mascot [27], fragmentation spectra obtained are assigned to putative peptide sequences and the assignments are then validated with programs like PeptideProphet [28] or Percolator [29]. The identification of these peptides allows for the identification of proteins present in the complex mixture.

Additionally, potential bioactive proteins and peptides can be characterized by protein-based bioinformatics tools. Such software includes programs to simulate *in-silico* proteolysis and to predict the physicochemical properties of the released peptides (i.e., antihypertensive, antimicrobial, immunomodulatory). Several bioactive peptide databases are available online such as APD3 [30], BioPep [31], BioPD [32], BioPepDB [33], CAMP [34], PPIP [35], starPepDB [36] and StraPep [37]. 

Therefore, the present work focuses for the first time on the global characterization of the jumbo squid (*Dosidicus gigas*) skin proteome using a shotgun proteomic approach. Meanwhile, a combination of different protein-based bioinformatics programs is carried out to determine potential bioactive peptides of this marine discard.

## 2. Results and Discussion

### 2.1. Jumbo Squid (Dosidicus gigas) Skin Proteome

A shotgun proteomics analysis for the jumbo squid (*Dosidicus gigas*) skin proteome is presented in this work, to our knowledge, for the first time. This repository was created merging a total of 6559 identified spectra (PSMs) from 1004 different peptides belonging to 219 different non-redundant annotated proteins from the different sample replicates (*n* = 4) (Supplementary Tables S1–S3). Table 1 summarizes the list of the non-redundant annotated proteins of the jumbo squid skin proteome (*n* = 219). This discovery stage was based on the LC-MS/MS analysis and SEQUEST-HT search of the tryptic digestions for the global protein extracts from the skin of each jumbo squid specimens studied (A–D replicates).

**Figure 1:**
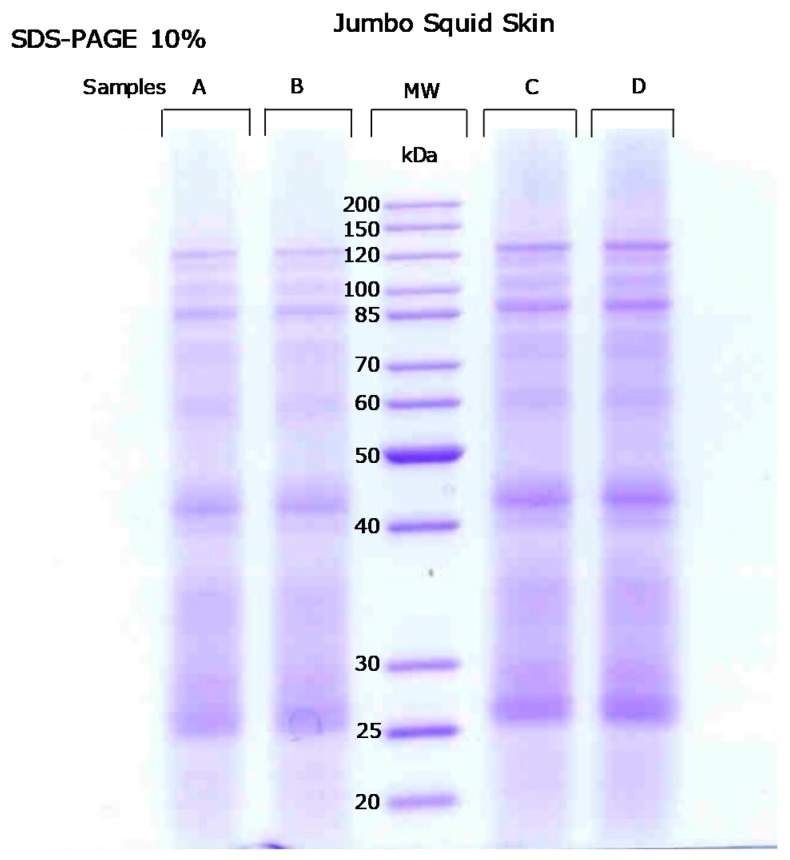
SDS-PAGE 10% profiles of the extracted proteins of jumbo squid skin samples (A–D replicates). MW denotes molecular weight.

Additionally, to visualize and corroborate the intact protein extraction of the jumbo squid skin fraction, complete protein extracts of the four replicates (A–D) were separated by SDS-PAGE 10% (Figure 1). This gel illustrates that all replicate extracts show the same protein weight distribution.

To our knowledge, this is the most comprehensive dataset of peptides and proteins for jumbo squid (*D. gigas*) skin identified to date. This valuable protein repository will add new and significant information to the universal public protein databases and could be very useful for new investigations of this marine by-product. Raw data and analyses outputs are publicly available in MassIVE data repository (https://massive.ucsd.edu/) (Reference: MSV000084702).

We need to take into account the difficulties and limitations of working with un-sequenced organisms as in the case of *D. gigas*. Thus, due to the fact that in the universal UniprotKB protein database only 40 different proteins for *D. gigas* are registered (Cytochrome c oxidase subunit 1, subunit 3; Cytochrome b; NADH-ubiquinone oxidoreductase chain 2, chain 4, chain 5; Cytochrome c oxidase subunit 2; ATP synthase subunit a; Histone H3; Chitin binding beak protein 1, 2, 3, 4; NADH dehydrogenase subunit 4L, subunit 2; ATP synthetase subunit 8; Paramyosin; Histidine rich beak protein 1, protein 2, protein 3; Suckerin-1, -2, -3, -4, -5, -6, -7, -8, -9, -10, -12, -13, -14, -15, -16, -17, -18, -20, -21; Symplectin/biotinidase-like protein), we decided to perform the protein identification using Proteome Discoverer 2.2 using a global database according to phylogenetic similarity for the class “Cephalopoda”. This class presents 40,780 entries, these including the 40 different proteins for *D. gigas* in order to increase the number of protein identifications. In Table 1, assignments for *D. gigas* protein are indicated in the first lines (Paramyosin and Symplectin/biotinidase-like protein). Many of the protein assignments are uncharacterized proteins (*n* = 109 proteins; *n* = 1393 PSMs) that may change with future Cephalopoda and *D. gigas* specific databases updates.

Thus, the final global dataset of the jumbo squid skin proteome was subsequently investigated by protein-based bioinformatics, like gene ontologies, pathways, network analyses and by prediction of potential bioactive peptides to gather more functional insights.

### 2.2. Functional Analysis: Gene Ontologies and Pathways Analysis

**Figure 2:**
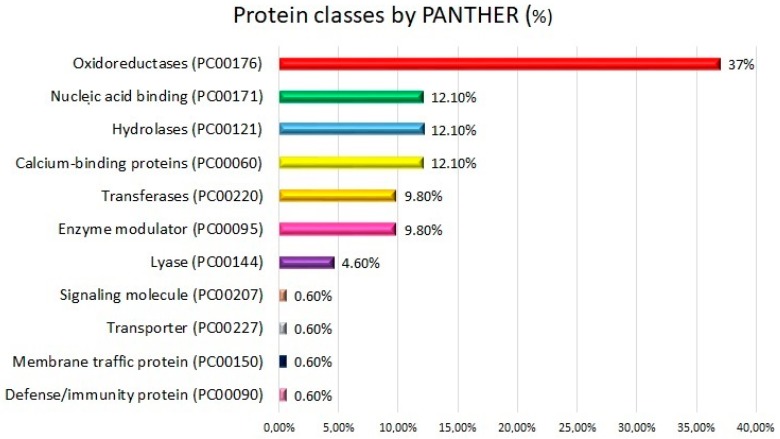
Protein classes of the jumbo squid skin proteome identified by shotgun proteomics and categorized by PANTHER (http://pantherdb.org/).

PANTHER analysis revealed the presence of 11 different protein classes in the jumbo squid skin proteome (Figure 2). The most prominent classes were oxidoreductases (37.0%), nucleic acid binding proteins (12.1%), hydrolases (12.1%), calcium-binding proteins (12.1%), transferases (9.8%), and enzyme modulator (9.8%). Thus, in the jumbo squid skin, oxidoreductases are mainly involved in the energetic metabolism, antioxidant defense and cephalopod coloration [38]. Another significant protein class is that of calcium-binding proteins, which are involved in muscle relaxation and nervous transmission in the marine skin species [39,40].

KEGG pathway analysis was carried out by comparing the input data with the background of the *Octopus bimaculoides* genome by DAVID version 6.8 program (https://david.ncifcrf.gov/home.jsp); this cephalopod species is the most phylogenetically closest included in DAVID software. KEGG showed that most of the identified proteins were involved in metabolic pathways (cysteine and methionine metabolism), endocytosis/phagosome, RNA transport, protein methylation, and calcium homeostasis (Table 2).

The study of functional domains by InterPro performed by DAVID software revealed that the top protein motifs corresponded to small GTP-binding protein domains, heat shock protein 70, small GTPase superfamily, proteasome, P-loop containing nucleoside triphosphate hydrolase and EF-hand-like domains (Table 3). These EF-hand domains corresponded to calcium-binding domains in concordance with the calcium homeostasis pathway discovered for the calcium-binding proteins, which correspond to 12.1% of the total jumbo squid skin proteome.

### 2.3. Network Analysis

**Figure 3:**
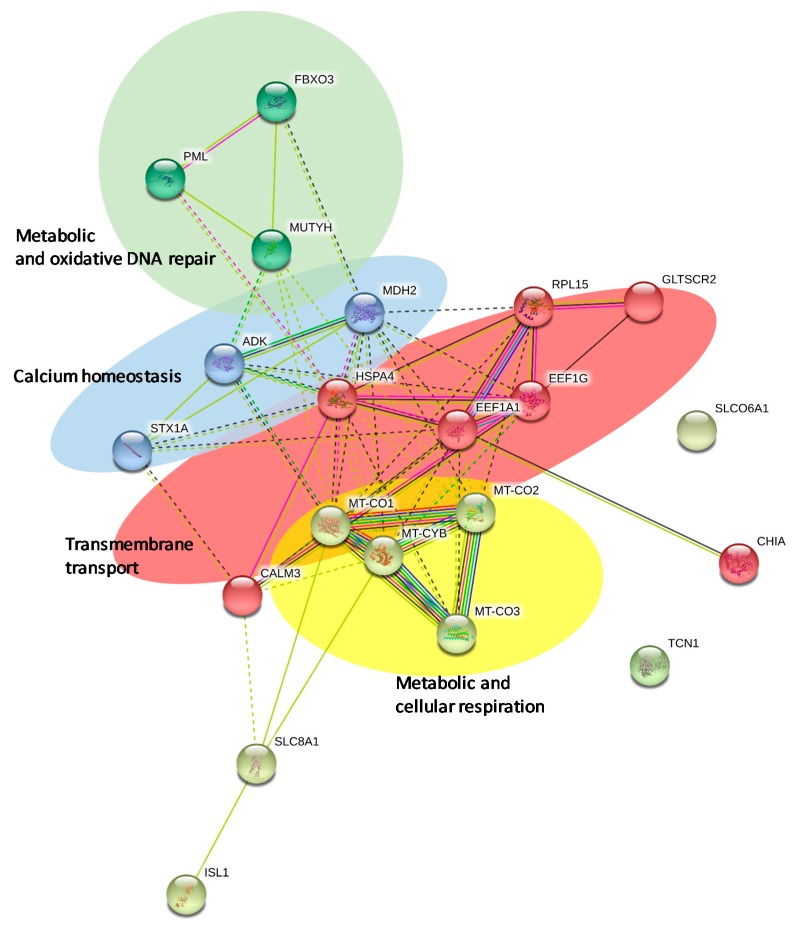
Protein network for the jumbo squid skin proteome using the STRING (v.11.0) software. Physical direct interactions are represented with continuous lines and functional interactions with interrupted lines.

Network analysis was created merging all the proteins identified for the jumbo squid skin proteome using the STRING software (v.11.0) (https://string-db.org/). A specific organism was not selected (organism Auto-detect) because the genome of *D. gigas* is not available in the STRING software. According to MCL inflation clustering (MCL = 3), 21 nodes (proteins) and 61 edges (interactions) were obtained (Figure 3). 

Physical direct interactions are represented with continuous lines and functional interactions with interrupted lines. The topological analysis of this network demonstrated mainly four different sub-networks. Two of them are relevant sub-networks implicated in metabolic and oxidative cellular respiration (Figure 3 in green and yellow). 

Other relevant sub-network is composed of three nodes and is referred as calcium homeostasis (Figure 3 in blue). The results of this sub-network are in concordance with one of the top protein classes categorized previously by PANTHER and DAVID (Figure 2 and Table 2). 

Other relevant sub-network is referred as transmembrane transport proteins (Figure 3, in red), as was obtained previously by PANTHER (Figure 2). 

Finally, this network represents to date the first most comprehensive interactomic map for the jumbo squid skin proteome.

### 2.4. Putative Bioactive Peptides

Bioactive peptides are inactive when they are part of parent protein, but become active when released due to the action of enzymes. Thus, bioactive peptides encrypted in the parent jumbo squid skin proteome (*n* = 219) were predicted using different *in-silico* software. Thus, protein hydrolysates with pepsin and trypsin were performed *in-silico* using the MS-Digest program. No missed cleavages and a minimum of six residues per peptide were selected as parameters. Thus, the predicted peptides after every enzymatic digestion (pepsin and trypsin) are presented in Supplementary Table S4.

The first enzymatic digestion using pepsin released a total of 5077 different peptides (6–39 amino acid residues). This enzyme cleaves the proteins at Phe, Tyr, Trp, and Leu residues in positions P1 and P1’ [41]. Compared with the most used and conventional BIOPEP database, no bioactive peptides were identified probably because none squid bioactive peptide is included in the database. However, by using PeptideRanker (http://distilldeep.ucd.ie/PeptideRanker/), the complete list of potential bioactive peptides was ranked using the *N*-to-1 neural network probability [42], which predicts the peptides that may be more bioactive (Supplementary Table S4). Among them, 18 peptides with a PeptideRanker score higher than 0.9 (7–30 amino acid residues) were selected as potential bioactive peptides (Table 4). The majority of the results corresponded to collagen ColAa proteins, hemocyanin subunit proteins and different uncharacterized proteins.

Regarding tryptic digestion, this enzyme predicted the release of a total of 8042 different peptides (6–45 amino acid residues) (Supplementary Table S4). This enzyme preferentially cleaves the proteins at Lys and Arg residues in position P1 except for the case in which Pro is found in position P1’ [41]. Using a PeptideRanker score higher than 0.9, a total of 73 tryptic peptides (7–30 amino acid residues) were selected as potential bioactive peptides (Table 5). The majority of such peptides corresponded to calcium-transporting ATPase, collagen ColAa proteins, hemocyanin proteins, myosin heavy chain, titin and different uncharacterized proteins.

It is known that the employment of collagenous residues obtained from jumbo squid skin after hydrolysis with pepsin exhibit a good gelatin gel-forming ability including the absence of color, opacity and high-puncture deformation [43]. The collagen alpha chains proteins determined in this study were characterized as belonging to type-I. Additionally, jumbo squid skin collagen was explored to enhance the anti-damage and anti-osteoporosis activity in osteoblast cells [44,45]. Thus, potential pepsin (PGDPGPVGRTGPMGL, RGPPGPPGL) and tryptic (GPPGIPGLPGPK, GPPGPPGLK, AGPPGFPGTPGPK) bioactive collagen peptides determined in this study may be used to stimulate the regeneration of joint cartilages in patients with chronic joint symptoms (Table 4 and Table 5). GELITA^®^ and CH-Alpha^®^ are examples of commercial products containing collagen hydrolysates.

Hemocyanins are the oxygen transporters of cephalopods and mollusks. These proteins play important immune-related roles as antimicrobial, antiviral, agglutinative and antitumor proliferation of cancer cells [46]. In fact, hemocyanin of marine mollusks (*Megathura crenulata* and *Concholepas concholepas*) has showed significant antitumor effects of breast, pancreas and prostate cancer cells [47,48]. Although, no previous studies are available related to the use of jumbo squid hemocyanin from a bioactive and immunotherapeutic point of view, it can be considered that the potential pepsin (KKPMMPF, PNQPMRPF, NDPMRPF, SDPMRPF) and tryptic (MVGYLGQALMALLLLALSNAALVR, FEPNPFFSGK, VACCLHGMPVFPHWHR, MATHWHSLLLFSLQLLVFTYATSDPTNIR, GSPIGVPYWDWTKPMK, TNFFFLALIATVWLGNAETETETSK, VFVGFLLHGFGSSAYATFDICNDAGECR, LNHLPLLCLAVILTLWMSGSNTVNGNLVR, VFAGFLFMGIK, VFAGFWFHGIK, VFGGFWLHGIK, TSFLFLAFVATSWFVYAVTASK) bioactive hemocyanin peptides determined in this study may be used in the future as an antitumor therapy for cancer cells (Table 4 and Table 5).

Calcium-transporting ATPase protein is an important regulator of the Ca^2+^ concentration in the cells and extracellular space. It is necessary for the cell signaling and for the nerve transmission of the squid axons [49]. Potential tryptic (FSDDYPGFF, FLQFQLTVNCVAVMVAFFGACIINDSPLK, FADAPFMK) bioactive calcium-transporting ATPase peptides determined in this study may be used in a future to investigate the *in vitro* axon stimulation (Table 5).

Myosin heavy chain is one of the major components of the muscle that participates in the muscle contraction as well as in a wide variety of non-muscular cells movements. Previous studies identified different ACE-inhibitory peptides from alcalase hydrolysis of a protein concentrate recovered from a cuttlefish (*Sepia officinalis*) industrial manufacturing effluent [17]. In fact, several potential bioactive peptides had a proline residue in one of the last positions of C-terminal which promotes enzyme binding (YQSGFIYTYSGLFCVAINPYR, YYSGLIYTYSGLFCVVVNPYK) [50] (Table 5). However, these results need to be further investigated because this is neither sufficient nor essential to confer bioactivity.

Titin (also known as connectin) is a giant protein that works as a molecular spring for the passive elasticity of tissues. The degradation of this protein is one of the major reasons for quality changes in fresh raw squid tissues [51]. Potential tryptic (DGSWQNLVTVLGCLKPQFVNLQR, GYPPPIISWYR) bioactive titin peptides determined in this study may be used as potential biomarkers of quality changes or processing time in squid products (Table 5).

The antimicrobial activity of jumbo squid skin crude pigments extracts has been recently demonstrated [52]. In the present work, antimicrobial peptides (AMPs) were identified using the CAMP (Collection of Anti-Microbial Peptides) database (http://www.bicnirrh.res.in/antimicrobial/) and applying the DAC score (Discriminate Analysis Classifier score) [34]. Table 4 and Table 5 show the potential anti-microbial bioactive peptides. A total of 16 pepsin peptides and 20 tryptic peptides with anti-microbial peptides were predicted. Among them, seven anti-microbial peptides (four pepsin and three tryptic) were encrypted in the hemocyanin parent protein (KKPMMPF, PNQPMRPF, NDPMRPF, SDPMRPF, VFAGFLFMGIK, VFAGFWFHGIK, VFGGFWLHGIK), two anti-microbial tryptic peptides in the collagen parent protein (GPPGIPGLPGPK, AGPPGFPGTPGPK), one anti-microbial tryptic peptide in the myosin heavy chain protein (NWQWWR) and one anti-microbial tryptic peptide in the titin protein (DGSWQNLVTVLGCLKPQFVNLQR).

All these potential bioactive peptides need to be validated by further bioactivity assays using synthetic versions of the peptides. Nevertheless, compared with the classical approaches, the bioinformatics methods are faster and lower-cost alternatives that predict and reduce the number of potential targets to be investigated. 

## 3. Materials and Methods 

### 3.1. Chemicals and Reagents

Bicinchoninic acid (BCA), dithiothreitol (DTT), sodium dodecyl sulphate (SDS), Tris-HCl, and the protease inhibitor phenylmethylsulphonyl fluoride (PMSF) were purchased from Sigma (St. Louis, MO, USA). Ammonium persulphate (APS), bromophenol blue and *N*,*N*,*N*′,*N*′-tetramethylethylenediamine (TEMED) were purchased from GE Healthcare Science (Uppsala, Sweden). Acrylamide and bis *N*,*N*′-methylene-bis-acrylamide were obtained from Bio-rad (Hercules, CA, USA). Glycerol was obtained from Merck (Darmstadt, Germany). Sequencing grade porcine trypsin was purchased from Promega (Madison, WI, USA). All other chemicals were reagent/analytical grade and water was purified using a Milli-Q system (Millipore, Billerica, MA, USA).

### 3.2. Jumbo Squids

Jumbo squid (*D. gigas*) specimens were harvested off the coast of Kino Bay, Mexico. Specimens were degutted and major beheaded on site, and the skins bagged and placed in alternate layers of ice-squid-ice in a portable cooler, and transported to the laboratory. Time between capture and arrival at the laboratory did not exceed 12 h. 

### 3.3. Skin Protein Samples

A total of 0.25 g of lyophilized jumbo squid skin were homogenized in 4 mL of lysis buffer (10 mM Tris-HCl buffer pH 7.2, 5 mM of PMSF) on ice for 6 cycles of 5 s pulses in a sonicator device (Werke, Germany). Samples were centrifuged at 40,000× g for 20 min at 4 °C in a J221-M centrifuge (Beckman, Palo Alto, CA, USA). The supernatant proteins were recovered and stored at −80 °C until used. Protein concentration in the protein extracts was determined by the bicinchoninic acid (BCA) method (Sigma Chemical Co., St. Louis, MI, USA). 

### 3.4. SDS-Polyacrylamide Gel Electrophoresis

Squid skin proteins were separated on 10% (*v*/*v*) polyacrylamide gels (acrylamide/*N*,*N*′-ethylene-bis-acrylamide, 200:1) with a stacking gel of 4% polyacrylamide. A total of 25 µg of proteins in Laemmli buffer were boiled for 5 min at 100 °C and separated per well in a Mini-PROTEAN 3 cell (Bio-Rad, Hercules, CA, USA). The running buffer consisted of an aqueous solution, composed by 1.44% (*w*/*v*) glycine, 0.67% Tris-base, and 0.1% SDS. Running conditions were 80 V for the first 20 min and then 120 V until the end of the electrophoresis. PageRuler unstained protein ladder was also used as molecular weight (*MW*) indicator (Thermo Fisher Scientific, San Jose, CA, USA).

Gels were stained overnight with Coomassie dye PhastGel Blue R-350 (GE Healthcare, Uppsala, Sweden). Scanned Coomassie-stained gels were analysed by means of the 1-d gel electrophoresis analysis software LabImage 1D (Kapelan Bio-Imaging Solutions, Halle, Germany).

### 3.5. In-Solution Protein Digestion with Trypsin

A total of 100 μg of jumbo squid skin protein extract were denatured in 8 M urea and then reduced with 5 mM TCEP (Pierce, Thermo Fisher Scientific) for 30 min at 37 °C. After alkylation with 50 mM iodoacetamide (Pierce, Thermo Fisher Scientific) in 25 mM ammonium bicarbonate pH 8.25 for 60 min at room temperature in dark, samples being diluted 4-fold with 25 mM ammonium bicarbonate pH 8.25 to decrease the urea concentration. Proteins were digested with trypsin (Promega) (1:100 protease-to-protein ratio) overnight at 37 °C.

### 3.6. Shotgun LC-MS/MS Analysis

Peptides were acidified with formic acid, cleaned on a C_18_ MicroSpin^TM^ column (The Nest Group, South-borough, MA) and analyzed by liquid chromatography-tandem mass spectrometry (LC-MS/MS) using a Proxeon EASY-nLC II liquid chromatography system (Thermo Fisher Scientific, San Jose, CA, USA) coupled to a LTQ-Orbitrap Elite mass spectrometer (Thermo Fisher Scientific). Peptide separation (1 µg) was done on a RP column (EASY-Spray column, 50 cm × 75 µm ID, PepMap C18, 2 µm particles, 100 Å pore size, Thermo Fisher Scientific) with a 10-mm pre-column (Accucore XL C18, Thermo Fisher Scientific) using 0.1% formic acid (mobile phase A) and 98% acetonitrile (98% ACN) with 0.1% formic acid (mobile phase B). A 120 min linear gradient from 5 to 35% B, at a flow rate of 300 nL min^−1^, was used. A spray voltage of 1.95 kV and a capillary temperature of 230 °C were used for ionization. The peptides were analyzed in positive mode (1 µscan; 400–1600 amu), followed by 10 data-dependent collision-induced dissociation (CID) MS/MS scans (1 µscans), using a normalized collision energy of 35% and an isolation width of 3 amu. Dynamic exclusion for 30 s after the second fragmentation event was applied and unassigned charged ions were excluded from the analysis.

A total of four replicates (*n* = 4) were analyzed independently.

### 3.7. Processing of the Mass Spectrometry Data 

All the MS/MS spectra were analyzed using SEQUEST-HT (Proteome Discoverer 2.2 package, Thermo Fisher Scientific) against the Cephalopoda UniProt/TrEMBL database (release 2018_11; 40,780 entries). The following restrictions were used: tryptic cleavage with up to 2 missed cleavage sites and tolerances of 0.8 Da for parent ions and 0.6 Da for MS/MS fragment ions. Carbamidomethylation of Cys (C*) was considered as a fixed modification. The permissible variable modifications were: methionine oxidation (Mox) and acetylation of the *N*-terminus of the protein (*N*-Acyl). The results were subjected to statistical analysis with the Percolator algorithm to keep a false discovery rate (FDR) below 1%.

### 3.8. Functional Gene Ontologies and Pathways Analysis

The final list of non-redundant protein IDs was submitted to PANTHER program (http://www.pantherdb.org/), for the classification based on two main types of annotations: protein class and biological process. A statistical significance of representation for the analysis was also provided.

KEGG pathway analysis was performed by comparing the input data with the background of the *Octopus bimaculoides* genome by DAVID version 6.8 (https://david.ncifcrf.gov/home.jsp). Functional domains by InterPro Motifs were also obtained using DAVID version 6.8 software.

### 3.9. Network Analysis

Network analysis was performed submitting the protein dataset to the STRING (Search Tool for the Retrieval of Interacting Genes) software (v.11.0) (http://stringdb.org/) [53]. This is a large database of known and predicted protein interactions. Proteins were represented with nodes and the interactions with continuous lines to represent direct interactions (physical), while indirect ones (functional) were presented by interrupted lines. To minimize false positives as well as false negatives, all interactions tagged as “low-confidence” (<0.4) in STRING software have been eliminated from the analysis. Cluster networks were created using the MCL inflation algorithm which is included in the STRING website and a value of 3 was selected for all the analyses.

### 3.10. Bioactive Peptides Prediction

Bioactive peptides encrypted in the parent jumbo squid skin proteome were predicted combining different *in-silico* protein hydrolysates using pepsin and trypsin enzymes. For that, all the proteolytic digestions were performed *in-silico* using the MS-Digest software, which is included in ProteinProspector v.5.24.0 website (http://prospector.ucsf.edu/prospector/mshome.htm).

To evaluate the results, all the potential peptides were ranked using the PeptideRanker software (http://bioware.ucd.ie/~testing/biowareweb/) using the *N*-to-1 neural network probability to predict which peptides can be more bioactive [42]. In addition, all the potential peptides were compared with previous databases that included known bioactive peptides, such as BIOPEP (http://www.uwm.edu.pl/biochemia/index.php/pl/biopep/) and CAMP (http://www.bicnirrh.res.in/antimicrobial/).

## 4. Conclusions

In this study, a shotgun proteomics strategy was applied for the first time for the characterization of the jumbo squid skin proteome. A total of 1004 different peptides belonging to 219 different proteins were identified. The final proteome compilation was investigated using different *in-silico* studies, including GO term enrichment, pathways and networks studies. The most prominent protein classes were oxidoreductases, calcium-binding proteins, hydrolases, nucleic acid binding, enzyme modulation, transferases involved in metabolic pathways (cysteine and methionine metabolism), endocytosis/phagosome, RNA transport, protein methylation, and calcium homeostasis. The first most comprehensive interactomic network map for the jumbo squid skin proteome was built up containing 21 nodes and 61 interactions. Most of the jumbo squid skin proteins were grouped under pathways and networks referring to metabolic and oxidative metabolism, calcium homeostasis, transmembrane transport and metabolic and cellular respiration. Moreover, potential valuable bioactive peptides were predicted after different *in-silico* digestions with pepsin and trysin. Antimicrobial, bioactive collagen peptides, antihypertensive, and antitumor properties were predicted to be present in the jumbo squid skin proteome. The integration of the global proteomics results and the bioinformatics analysis of the jumbo squid skin proteome show a comprehensive knowledge of this fishery discard and provide potential bioactive peptides of this marine by-product.

## Figures and Tables

**Table 1:** Jumbo squid (*Dosidicus gigas*) skin proteome (FDR < 1%). See Supplementary Tables S1–S3 for complete information.

*N*	Accession	Description	Gene	Uni. Pep.	PSM	Cov. (%)
1	A0A1Y1DCG9	Paramyosin OS = *Dosidicus gigas*	DgPm	17	46	22
2	A0A2Z5EQ31	Symplectin/biotinidase-like protein OS = *Dosidicus gigas*	sympp	1	2	3
3	A0A0P0UX03	Hemocyanin subunit 1 OS = *Todarodes pacificus*	Tphcy	116	3007	38
4	A0A077B1P8	Hemocyanin subunit 2 OS = *Euprymna scolopes*	HCY2	10	1608	24
5	A0A077B6R8	Hemocyanin subunit 1 OS = *Euprymna scolopes*	HCY1	13	1437	19
6	T2F8L5	Hemocyanin OS = *Sepiella maindroni*	HCY1	8	1544	18
7	W6CNR9	Hemocyanin subunit 3 OS = *Sepia officinalis*	HCY3	10	1035	13
8	A0A1Q2SJF4	Hemocyanin-like protein OS = *Uroteuthis edulis*	hc	8	746	14
9	F1ADJ4	Myosin heavy chain OS = *Todarodes pacificus*	MYH	16	456	15
10	I0JGT9	Actin I OS = *Sepia officinalis*	ACTI	11	202	53
11	G4V4Y8	Myosin heavy chain isoform C OS = *Doryteuthis pealeii*	MYH	3	411	12
12	A4D0I0	Hemocyanin subunit 1 OS = *Todarodes pacificus*	Tphcy	6	174	50
13	A0A0P0UX01	Hemocyanin subunit2 OS = *Todarodes pacificus*	Tphcy	4	171	51
14	A0A0L8G4B4	Uncharacterized protein OS = *Octopus bimaculoides*	OCBIM_22000685mg	27	53	13
15	V6A729	Myosin heavy chain isoform A OS = *Octopus bimaculoides*	MYH	2	348	8
16	Q2V0V2	Tropomyosin OS = *Todarodes pacificus*	tp-tm	27	127	46
17	A0A0L8GFI1	Spectrin beta chain OS = *Octopus bimaculoides*	OCBIM_22034275mg	24	72	12
18	I7H9I6	Haemocyanin OS = *Nautilus pompilius*	hc	1	532	5
19	A0A075IT96	Heat shock protein 70 OS = *Sepiella maindroni*	HSP70	3	59	23
20	A0A0L8HMH4	Uncharacterized protein OS = *Octopus bimaculoides*	OCBIM_22011261mg	12	35	3
21	E7CLR5	Hemocyanin (Fragment) OS = *Spirula spirula*	HCY1	1	315	12
22	A0A0L8IA52	Uncharacterized protein OS = *Octopus bimaculoides*	OCBIM_22026555mg	1	49	18
23	A0A0L8GPG8	Uncharacterized protein OS = *Octopus bimaculoides*	OCBIM_22030693mg	11	59	17
24	A0A0L8FFZ3	Uncharacterized protein OS = *Octopus bimaculoides*	OCBIM_22022789mg	2	394	30
25	A0A0L8H027	Uncharacterized protein OS = *Octopus bimaculoides*	OCBIM_22024964mg	8	48	5
26	A0A0L8G0V9	Uncharacterized protein OS = *Octopus bimaculoides*	OCBIM_22003270mg	6	32	16
27	Q06270	Intermediate filament protein OS = *Nototodarus sloanii*	OCBIM_22025455mg	9	38	18
28	Q76EJ2	Cathepsin D OS = *Todarodes pacificus*	tpaD	9	49	22
29	P08052	Myosin regulatory light chain LC-2, mantle muscle OS = *Todarodes pacificus*	MYL	8	23	50
30	A0A0L8HC80	Uncharacterized protein OS = *Octopus bimaculoides*	OCBIM_22017953mg	8	16	5
31	A0A0L8G3E9	Uncharacterized protein OS = *Octopus bimaculoides*	OCBIM_22001601mg	1	31	11
32	P30842	Omega-crystallin OS = *Nototodarus sloanii*	N/A	5	22	9
33	Q68LN1	Filamin OS = *Euprymna scolopes*	OCBIM_22031719mg	4	20	34
34	A0A0L8FU30	Uncharacterized protein OS = *Octopus bimaculoides*	OCBIM_22007941mg	1	12	33
35	A0A0L8I9I4	Uncharacterized protein OS = *Octopus bimaculoides*	OCBIM_22028792mg	1	18	22
36	A0A0L8FNC4	Uncharacterized protein OS = *Octopus bimaculoides*	OCBIM_22013362mg	5	12	4
37	Q6E216	Tropomysin-like protein OS = *Todarodes pacificus*	ATRP	5	9	26
38	A0A0L8HDP4	Uncharacterized protein OS = *Octopus bimaculoides*	OCBIM_22016840mg	3	27	5
39	A0A0L8FVD0	Uncharacterized protein OS = *Octopus bimaculoides*	OCBIM_22007411mg	4	15	27
40	A0A0L8GWE3	Uncharacterized protein OS = *Octopus bimaculoides*	OCBIM_22026600mg	3	13	12
41	A0A0L8HKK9	Fructose-bisphosphate aldolase OS = *Octopus bimaculoides*	OCBIM_22013272mg	3	21	7
42	A0A0L8FP56	Uncharacterized protein OS = *Octopus bimaculoides*	OCBIM_22013360mg	1	16	3
43	G1CW44	Triosephosphate isomerase OS = *Enteroctopus dofleini*	OCBIM_22037419mg	1	27	11
44	G1CW45	Triosephosphate isomerase OS = *Euprymna scolopes*	OCBIM_22037419mg	1	8	19
45	A0A0L8GN79	Uncharacterized protein OS = *Octopus bimaculoides*	OCBIM_22030767mg	2	11	9
46	A0A0L8FZT7	Protein disulfide-isomerase OS = *Octopus bimaculoides*	OCBIM_22003356mg	3	17	8
47	A0A0L8H0K3	Uncharacterized protein OS = *Octopus bimaculoides*	OCBIM_22024969mg	3	8	7
48	A0A0L8GNQ0	Uncharacterized protein OS = *Octopus bimaculoides*	OCBIM_22030666mg	2	5	10
49	A0A0L8IA72	Uncharacterized protein OS = *Octopus bimaculoides*	OCBIM_22025549mg	4	8	7
50	A0A0L8IAK7	Uncharacterized protein OS = *Octopus bimaculoides*	OCBIM_22025100mg	5	9	1
51	A0A0L8HDG9	Uncharacterized protein OS = *Octopus bimaculoides*	OCBIM_22017348mg	3	4	20
52	Q86DP6	Malate dehydrogenase (Fragment) OS = *Sepia officinalis*	Mdh	3	7	11
53	P05945	Myosin catalytic light chain LC-1, mantle muscle OS = *Todarodes pacificus*	MYL	2	6	19
54	A0A0L8GQL2	Tubulin beta chain OS = *Octopus bimaculoides*	OCBIM_22029847mg	3	8	8
55	A0A0L8HMP5	Uncharacterized protein OS = *Octopus bimaculoides*	OCBIM_22011994mg	3	10	16
56	A0A0L8IAD9	Uncharacterized protein OS = *Octopus bimaculoides*	OCBIM_22025091mg	3	3	6
57	A0A0L8FJA0	Uncharacterized protein OS = *Octopus bimaculoides*	OCBIM_22017780mg	2	6	3
58	A0A0L8G425	Adenosylhomocysteinase OS = *Octopus bimaculoides*	OCBIM_22000532mg	3	6	7
59	A0A0L8FXP2	Uncharacterized protein OS = *Octopus bimaculoides*	OCBIM_22004658mg	3	3	5
60	A0A0L8I198	Uncharacterized protein OS = *Octopus bimaculoides*	OCBIM_22039192mg	3	8	19
61	A0A0L8I871	Uncharacterized protein OS = *Octopus bimaculoides*	OCBIM_22028797mg	2	7	18
62	A0A2S1FRU3	Elongation factor 1-alpha OS = *Callistoctopus minor*	EEF1A1	4	6	7
63	A0A0L8FFD9	Uncharacterized protein OS = *Octopus bimaculoides*	OCBIM_22023810mg	2	3	2
64	A0A0L8I874	Uncharacterized protein OS = *Octopus bimaculoides*	OCBIM_22028979mg	2	6	16
65	A0A0L8FK19	Tubulin alpha chain OS = *Octopus bimaculoides*	OCBIM_22016917mg	2	5	3
66	A0A0K0WTY3	Arginine kinase OS = *Sepia pharaonis*	AK	4	7	7
67	A0A0L8GXA0	Glucosamine-6-phosphate isomerase OS = *Octopus bimaculoides*	OCBIM_22026276mg	1	3	9
68	F8V2T7	Sodium/potassium-transporting ATPase subunit alpha OS = *Bathypolypus arcticus*	OCBIM_22028074mg	2	4	2
69	A0A0L8H4W4	Proteasome subunit alpha type OS = *Octopus bimaculoides*	OCBIM_22022293mg	2	3	10
70	A0A0L8GSZ5	Histone H4 OS = *Octopus bimaculoides*	OCBIM_22029078mg	2	5	10
71	A0A0L8GDJ1	Uncharacterized protein OS = *Octopus bimaculoides*	OCBIM_22035502mg	2	4	6
72	A0A159BRC2	ColAa OS = *Sepia pharaonis*	N/A	2	6	1
73	A0A0L8FIB5	Uncharacterized protein OS = *Octopus bimaculoides*	OCBIM_22020215mg	1	2	5
74	A0A0L8G4U5	Uncharacterized protein OS = *Octopus bimaculoides*	OCBIM_22000359mg	2	4	6
75	Q9NL93	G protein a subunit o class OS = *Octopus vulgaris*	OvGao	2	5	6
76	A0A0L8IG11	Uncharacterized protein OS = *Octopus bimaculoides*	OCBIM_22004528mg	2	8	11
77	A0A0L8GG89	Proteasome subunit alpha OS = *Octopus bimaculoides*	OCBIM_22033871mg	2	3	9
78	A0A0L8H716	Uncharacterized protein OS = *Octopus bimaculoides*	OCBIM_22020867mg	2	12	11
79	A0A0S1U346	Triosephosphate isomerase OS = *Amphioctopus fangsiao*	OCBIM_22037419mg	1	3	18
80	A0A0L8H4E7	Uncharacterized protein OS = *Octopus bimaculoides*	OCBIM_22022663mg	2	4	6
81	A0A0L8I919	Uncharacterized protein OS = *Octopus bimaculoides*	OCBIM_22027793mg	1	7	5
82	A0A0L8HN83	Uncharacterized protein OS = *Octopus bimaculoides*	OCBIM_22010679mg	1	1	3
83	A0A0L8ICB5	Uncharacterized protein OS = *Octopus bimaculoides*	OCBIM_22019476mg	2	4	4
84	A0A0L8FMD3	Uncharacterized protein OS = *Octopus bimaculoides*	OCBIM_22014986mg	1	2	4
85	A0A0L8H0E1	Sorting nexin OS = *Octopus bimaculoides*	OCBIM_22024936mg	1	5	3
86	A0A0L8IA39	Tubulin alpha chain OS = *Octopus bimaculoides*	OCBIM_22026381mg	1	2	3
87	A0A0L8IG73	Malic enzyme OS = *Octopus bimaculoides*	OCBIM_22004207mg	1	1	3
88	A0A0L8H635	Uncharacterized protein OS = *Octopus bimaculoides*	OCBIM_22021483mg	1	2	8
89	A0A0L8GYT6	Uncharacterized protein OS = *Octopus bimaculoides*	OCBIM_22026168mg	1	3	10
90	A0A0L8GFD5	Uncharacterized protein OS = *Octopus bimaculoides*	OCBIM_22034343mg	1	2	3
91	A0A0L8HKN4	Ornithine aminotransferase OS = *Octopus bimaculoides*	OCBIM_22012517mg	1	4	3
92	A0A0L8G0I6	Uncharacterized protein OS = *Octopus bimaculoides*	OCBIM_22003454mg	2	2	4
93	A0A0L8HE61	AP complex subunit beta OS = *Octopus bimaculoides*	OCBIM_22016805mg	1	1	1
94	A0A0L8HMS6	Uncharacterized protein OS = *Octopus bimaculoides*	OCBIM_22011048mg	1	3	3
95	A0A0L8FWD6	Calcium-transporting ATPase OS = *Octopus bimaculoides*	OCBIM_22006279mg	2	6	2
96	A0A0L8GP54	Uncharacterized protein OS = *Octopus bimaculoides*	OCBIM_22030838mg	1	2	7
97	A0A0L8G9P1	Uncharacterized protein OS = *Octopus bimaculoides*	OCBIM_22037676mg	1	4	9
98	A0A0L8HTA6	Uncharacterized protein OS = *Octopus bimaculoides*	OCBIM_22007620mg	1	4	6
99	A0A0L8IAN9	Uncharacterized protein OS = *Octopus bimaculoides*	OCBIM_22025097mg	1	1	8
100	A0A0L8HCU8	Uncharacterized protein OS = *Octopus bimaculoides*	OCBIM_22018310mg	1	3	2
101	A0A0A7NZU2	Putative chitotriosidase OS = *Euprymna scolopes*	Chia	1	1	4
102	A0A0L8G3Z0	Uncharacterized protein OS = *Octopus bimaculoides*	OCBIM_22000581mg	1	3	4
103	A0A0L8I836	Uncharacterized protein OS = *Octopus bimaculoides*	OCBIM_22028993mg	1	3	3
104	A0A0L8IDP3	Uncharacterized protein OS = *Octopus bimaculoides*	OCBIM_22014847mg	1	1	4
105	A0A0L8FZ08	Uncharacterized protein OS = *Octopus bimaculoides*	OCBIM_22004461mg	1	1	1
106	A0A0L8GZM9	Uncharacterized protein OS = *Octopus bimaculoides*	OCBIM_22025211mg	1	4	2
107	A0A193PD55	Chitinase OS = *Todarodes pacificus*	TpChi	1	2	2
108	Q8IS80	60S acidic ribosomal protein OS = *Euprymna scolopes*	OCBIM_22035130mg	1	3	19
109	A0A0L8FQ90	Serine/threonine-protein phosphatase OS = *Octopus bimaculoides*	OCBIM_22011907mg	1	1	4
110	A0A0L8FIY8	Uncharacterized protein OS = *Octopus bimaculoides*	OCBIM_22018177mg	1	3	13
111	A0A0L8I107	Uncharacterized protein OS = *Octopus bimaculoides*	OCBIM_22039276mg	1	2	4
112	A0A0L8G4M6	Uncharacterized protein OS = *Octopus bimaculoides*	OCBIM_22000216mg	1	2	0
113	A0A0L8GLC5	Uncharacterized protein OS = *Octopus bimaculoides*	OCBIM_22031874mg	1	3	8
114	A0A0L8HDX1	Superoxide dismutase OS = *Octopus bimaculoides*	OCBIM_22016770mg	1	2	6
115	A0A0L8HU31	Uncharacterized protein OS = *Octopus bimaculoides*	OCBIM_22005978mg	1	2	3
116	Q8SWQ7	Non-muscle myosin II heavy chain OS = *Doryteuthis pealeii*	MYH	1	1	1
117	B8Q2 × 2	G alpha q subunit OS = *Euprymna scolopes*	COI	1	1	5
118	A0A0L8G1S2	Uncharacterized protein OS = *Octopus bimaculoides*	OCBIM_22001882mg	1	1	3
119	A0A0L8HAV5	Uncharacterized protein OS = *Octopus bimaculoides*	OCBIM_22019117mg	1	1	7
120	A0A0L8IDX1	Uncharacterized protein OS = *Octopus bimaculoides*	OCBIM_22013485mg	1	4	4
121	A0A0L8GRX5	Histone H2B OS = *Octopus bimaculoides*	OCBIM_22029075mg	1	1	6
122	A0A0L8FS75	Proteasome subunit alpha type OS = *Octopus bimaculoides*	OCBIM_22010113mg	1	2	4
123	A0A0L8FRK2	Uncharacterized protein OS = *Octopus bimaculoides*	OCBIM_22010655mg	1	2	6
124	A0A0L8GZX1	Uncharacterized protein OS = *Octopus bimaculoides*	OCBIM_22025682mg	1	5	1
125	A0A0L8G456	Uncharacterized protein OS = *Octopus bimaculoides*	OCBIM_22000796mg	1	1	6
126	A0A0L8FF63	Uncharacterized protein OS = *Octopus bimaculoides*	OCBIM_22024380mg	1	1	10
127	A0A0L8H8U9	Uncharacterized protein OS = *Octopus bimaculoides*	OCBIM_22020735mg	1	1	5
128	A0A0L8I5N4	Uncharacterized protein OS = *Octopus bimaculoides*	OCBIM_22033390mg	1	2	3
129	A0A0L8I398	Uncharacterized protein OS = *Octopus bimaculoides*	OCBIM_22037157mg	1	1	11
130	A0A0L8GP93	Nicotinamide-nucleotide adenylyltransferase OS = *Octopus bimaculoides*	OCBIM_22030204mg	1	1	6
131	A0A0L8IIH3	Uncharacterized protein OS = *Octopus bimaculoides*	OCBIM_22025740mg	1	3	0
132	A0A0L8GZD4	Uncharacterized protein OS = *Octopus bimaculoides*	OCBIM_22025455mg	1	1	1
133	A0A0L8HQW9	Uncharacterized protein OS = *Octopus bimaculoides*	OCBIM_22008430mg	1	4	2
134	A0A0L8G2Z7	Small ubiquitin-related modifier OS = *Octopus bimaculoides*	OCBIM_22001102mg	1	1	11
135	A0A0L8G8L3	Uncharacterized protein OS = *Octopus bimaculoides*	OCBIM_22038063mg	1	2	2
136	O46345	S-syntaxin OS = *Doryteuthis pealeii*	STX1	1	1	3
137	A0A0L8GDD2	Uncharacterized protein OS = *Octopus bimaculoides*	OCBIM_22036000mg	1	1	2
138	C4N147	Sodium/calcium exchanger regulatory protein 1 OS = *Doryteuthis pealeii*	SLC8A1	1	4	7
139	A0A0L8FJE4	Uncharacterized protein OS = *Octopus bimaculoides*	OCBIM_22017696mg	1	2	2
140	A0A0L8I067	Kinesin-like protein OS = *Octopus bimaculoides*	OCBIM_22000619mg	1	1	1
141	A0A0L8FYB6	Uncharacterized protein OS = *Octopus bimaculoides*	OCBIM_22005155mg	1	1	1
142	A0A0L8GUV0	Serine/threonine-protein phosphatase OS = *Octopus bimaculoides*	OCBIM_22027338mg	1	1	2
143	A0A0L8GJ12	Uncharacterized protein OS = *Octopus bimaculoides*	OCBIM_22032700mg	1	2	1
144	A0A0L8GLG2	Uncharacterized protein OS = *Octopus bimaculoides*	OCBIM_22032112mg	1	1	1
145	A0A0L8GY97	Uncharacterized protein OS = *Octopus bimaculoides*	OCBIM_22026356mg	1	2	2
146	Q27Q56	Hemocyanin subunit 2 OS = *Sepia officinalis*	HCY2	1	961	7
147	A0A161HPY5	Actin OS = *Crassostrea brasiliana*	ACTI	3	96	38
148	D2YZ90	Beta actin OS = *Idiosepius paradoxus*	ACTI	2	95	37
149	K1QFR9	Spectrin beta chain OS = *Crassostrea gigas*	CGI_10013845	1	34	4
150	C1KC83	Heat shock cognate protein 70 OS = *Haliotis diversicolor*	HSP70	1	37	16
151	A0A2C9K1T4	Uncharacterized protein OS = *Biomphalaria glabrata*	106078167	1	68	13
152	A0A0B7B7H2	Uncharacterized protein OS = *Arion vulgaris*	ORF162822	1	40	11
153	A0A2T7NLR4	Uncharacterized protein OS = *Pomacea canaliculata*	C0Q70_17913	1	18	5
154	K1RH58	Alpha-actinin, sarcomeric OS = *Crassostrea gigas*	CGI_10003110	1	43	10
155	A0A2P1H676	Heat shock protein 70 OS = *Diplodon chilensis*	HSP70	1	36	12
156	K1PMY9	Calmodulin OS = *Crassostrea gigas*	CGI_10006482	1	22	13
157	A0A2T7NGU8	Uncharacterized protein OS = *Pomacea canaliculata*	C0Q70_18553	5	12	29
158	Q564J1	Haemocyanin OS = *Aplysia californica*	hc	2	927	2
159	A0A2T7NV41	Uncharacterized protein OS = *Pomacea canaliculata*	C0Q70_15545	4	18	25
160	E7DS67	Actin (Fragment) OS = *Gonospira metablata*	ACTI	1	38	18
161	K1RBG6	Actin-1/3 OS = *Crassostrea gigas*	CGI_10017112	1	39	8
162	P02595	Calmodulin OS = *Patinopecten sp.*	CAM	1	16	30
163	V6A758	Myosin heavy chain isoform C OS = *Sepia officinalis*	MYH	1	17	16
164	A0A0B7BLG3	Uncharacterized protein OS = *Arion vulgaris*	ORF192624	3	23	2
165	K1PPW8	Coatomer subunit beta OS = *Crassostrea gigas*	CGI_10006442	2	8	7
166	A0A210R0F2	Fructose-bisphosphate aldolase OS = *Mizuhopecten yessoensis*	KP79_PYT16607	2	8	6
167	A0A2T7PZW7	Uncharacterized protein OS = *Pomacea canaliculata*	C0Q70_01565	1	6	1
168	A0A0B7B4N1	Uncharacterized protein OS = *Arion vulgaris*	ORF158201	1	10	4
169	A0A210QY92	Coatomer subunit beta’ OS = *Mizuhopecten yessoensis*	KP79_PYT21841	1	5	5
170	V3ZPS1	Uncharacterized protein OS = *Lottia gigantea*	LOTGIDRAFT_222012	2	9	12
171	E3VWM3	Fructose-bisphosphate aldolase OS = *Meretrix meretrix*	FBA	1	20	4
172	A0A2T7PSV4	Uncharacterized protein OS = *Pomacea canaliculata*	C0Q70_03483	2	10	11
173	A0A0B7AZA8	Uncharacterized protein OS = *Arion vulgaris*	ORF148015	2	10	19
174	K7WKX6	Fructose-bisphosphate aldolase OS = *Haliotis rufescens*	FBA	1	3	9
175	A0A2T7NF32	Uncharacterized protein OS = *Pomacea canaliculata*	C0Q70_20261	1	5	4
176	A0A2T7NMW4	Uncharacterized protein OS = *Pomacea canaliculata*	C0Q70_18325	2	4	5
177	K1QZU8	Calcium-transporting ATPase OS = *Crassostrea gigas*	CGI_10023684	1	2	1
178	A0A0L8IAE8	Uncharacterized protein OS = *Octopus bimaculoides*	OCBIM_22025089mg	1	2	8
179	A0A2C9KC89	Uncharacterized protein OS = *Biomphalaria glabrata*	106056965	2	5	3
180	A0A210R746	Ras-related protein Rab-6A OS = *Mizuhopecten yessoensis*	KP79_PYT20147	1	9	11
181	A0A0B6Z4Q3	Uncharacterized protein OS = *Arion vulgaris*	ORF48472	2	12	8
182	A0A2T7PZP4	Uncharacterized protein OS = *Pomacea canaliculata*	C0Q70_01513	1	4	3
183	A0A2C9JIZ4	Uncharacterized protein OS = *Biomphalaria glabrata*	106056849	1	9	13
184	K1PTH4	ADP-ribosylation factor OS = *Crassostrea gigas*	CGI_10020174	1	2	1
185	Q6PTL0	Triosephosphate isomerase OS = *Nucula proxima*	OCBIM_22037419mg	1	5	6
186	A0A2C9JZR8	Uncharacterized protein OS = *Biomphalaria glabrata*	106074442	1	2	2
187	A0A2C9JIA9	Uncharacterized protein OS = *Biomphalaria glabrata*	106056539	1	6	4
188	A0A385NHM7	Glutathione S-transferase OS = *Tegillarca granosa*	GST	1	8	5
189	A0A210QUP5	Malic enzyme OS = *Mizuhopecten yessoensis*	KP79_PYT06884	1	1	3
190	V3YXF9	Adenosylhomocysteinase OS = *Lottia gigantea*	LOTGIDRAFT_184532	1	2	3
191	A0A210QGP4	Chitotriosidase-1 OS = *Mizuhopecten yessoensis*	KP79_PYT06201	1	1	3
192	A0A210QHE1	Adenosylhomocysteinase OS = *Mizuhopecten yessoensis*	KP79_PYT14445	1	4	3
193	A0A210PIA6	Ornithine aminotransferase OS = *Mizuhopecten yessoensis*	KP79_PYT16913	1	3	3
194	K1QQB6	40S ribosomal protein S14 OS = *Crassostrea gigas*	CGI_10011151	1	4	9
195	A0A2C9KEN8	Tubulin alpha chain OS = *Biomphalaria glabrata*	106069694	1	2	3
196	A0A2T7PWT6	Serine/threonine-protein phosph OS = *Pomacea canaliculata*	C0Q70_00460	1	1	3
197	A0A0B7AJW7	Fructose-bisphosphate aldolase OS = *Arion vulgaris*	ORF124546	1	8	4
198	A0A2C9L7N6	Uncharacterized protein OS = *Biomphalaria glabrata*	106080319	1	49	4
199	A0A210QTZ1	Peptidyl-prolyl cis-trans OS = *Mizuhopecten yessoensis*	KP79_PYT00632	1	2	6
200	A0A2I7M8C2	Go protein alpha subunit OS = *Argopecten irradians*	N/A	1	4	3
201	K1R2G8	Titin OS = *Crassostrea gigas*	CGI_10016808	1	2	0
202	K1QVD7	Neuronal acetylcholine receptor subunit non-alpha-2 OS = *Crassostrea gigas*	CGI_10016138	1	2	1
203	K1Q7G5	Ficolin-2 OS = *Crassostrea gigas*	CGI_10026202	1	2	3
204	A0A2C9K9W9	Uncharacterized protein OS = *Biomphalaria glabrata*	106068683	1	1	1
205	A0A0B6ZP87	Uncharacterized protein OS = *Arion vulgaris*	ORF71130	1	3	4
206	V4AP92	Elongation factor 1-alpha OS = *Lottia gigantea*	LOTGIDRAFT_239271	1	2	2
207	A0A2T7PU69	Uncharacterized protein OS = *Pomacea canaliculata*	C0Q70_03920	1	4	4
208	V3ZN51	Staphylococcal nuclease domain-cont. OS = *Lottia gigantea*	LOTGIDRAFT_235720	1	3	1
209	A0A2T7PSF5	Uncharacterized protein OS = *Pomacea canaliculata*	C0Q70_03333	1	2	0
210	K1PQD4	Phosphoglucomutase-1 OS = *Crassostrea gigas*	CGI_10011818	1	1	2
211	A0A0B7BF17	Uncharacterized protein OS = *Arion vulgaris*	ORF179770	1	3	2
212	A0A2T7Q0W0	Uncharacterized protein OS = *Pomacea canaliculata*	C0Q70_01928	1	1	3
213	A0A0L8I692	Uncharacterized protein OS = *Octopus bimaculoides*	OCBIM_22034637mg	1	4	19
214	K1PQ79	Copine-3 OS = *Crassostrea gigas*	CGI_10011897	1	3	1
215	K1PWB9	EH domain-containing protein 1 OS = *Crassostrea gigas*	CGI_10005813	1	1	4
216	A0A2T7Q016	Uncharacterized protein OS = *Pomacea canaliculata*	C0Q70_01636	1	1	2
217	V4AKV4	Calcium-transporting ATPase OS = *Lottia gigantea*	LOTGIDRAFT_208914	1	3	1
218	A0A2T7NL99	Proteasome subunit beta OS = *Pomacea canaliculata*	C0Q70_17739	1	2	4
219	A0A0L8HWW8	Uncharacterized protein OS = *Octopus bimaculoides*	OCBIM_22003772mg	1	2	2

*N* (Identification Number); FDR (False Discovery Rate); Uni. Pep. (Unique Peptides); PSMs (Peptide Spectrum Matches); Cov. (Protein Coverage).

**Table 2:** KEGG pathway analysis of the jumbo squid skin proteome by DAVID.

KEGG Pathway	*p*-Value
Metabolic pathways (cysteine and methionine metabolism)	4.53 × 10^−4^
Endocytosis/phagosome	1.05 × 10^−2^
RNA transport	2.24 × 10^−2^
Protein methylation	3.46 × 10^−2^
Calcium homeostasis	1.00 × 10^−1^

**Table 3:** Functional InterPro motifs by DAVID.

InterPro Motifs	*p*-Value
Small GTP-binding protein domain	3.1 × 10^−4^
Heat shock protein 70, conserved site	8.5 × 10^−4^
Small GTPase superfamily	8.6 × 10^−4^
Proteasome, alpha-subunit, N-terminal domain	1.3 × 10^−3^
P-loop containing nucleoside triphosphate hydrolase	8.3 × 10^−3^
EF-hand-like domain	2.9 × 10^−2^
Ubiquitin	3.4 × 10^−2^

**Table 4:** Selected potential bioactive peptides of the jumbo squid skin proteome predicted by *in-silico* digestions with pepsin.

Proteins	Peptides	PeptideRanker Score	Anti-Microbial Peptide (AMP)	Discriminant Score for AMP
ADP-ribosylation factor OS = *Crassostrea gigas*	SPSPKQMVSCPVCGL	0.915222	Non-AMP	0.043
Collagen ColAa OS = *Sepia pharaonis*	PGDPGPVGRTGPMGL	0.934847	Non-AMP	0.003
Collagen ColAa OS = *Sepia pharaonis*	RGPPGPPGL	0.912657	Non-AMP	0.030
Heat shock protein 70 OS = *Sepiella maindroni*	GGMPGGMPGGMPGGMPNF	0.92432	AMP	0.504
Hemocyanin OS = *Sepiella maindroni*	KKPMMPF	0.932566	AMP	0.978
Hemocyanin OS = *Sepiella maindroni*	PNQPMRPF	0.920777	AMP	0.983
Hemocyanin subunit 1 OS = *Todarodes pacificus*	NDPMRPF	0.923312	AMP	0.795
Hemocyanin subunit 2 OS = *Sepia officinalis*	SDPMRPF	0.938433	AMP	0.879
Uncharacterized protein OS = *Octopus bimaculoides*	CPCMGRF	0.985441	AMP	0.622
Uncharacterized protein OS = *Octopus bimaculoides*	GGPPGMPPF	0.973279	Non-AMP	0.208
Uncharacterized protein OS = *Octopus bimaculoides*	GRCVMCNCNKHSSTCDPQTGKCVNCQHNTL	0.969319	Non-AMP	0.238
Uncharacterized protein OS = *Octopus bimaculoides*	GSCVPCNCNGF	0.952459	AMP	0.745
Uncharacterized protein OS = *Octopus bimaculoides*	QPPQCCPSKGGSF	0.943546	AMP	0.687
Uncharacterized protein OS = *Octopus bimaculoides*	GSWGNGNRW	0.915802	Non-AMP	0.403
Uncharacterized protein OS = *Octopus bimaculoides*	PPPSKRF	0.911736	AMP	0.983
Uncharacterized protein OS = *Biomphalaria glabrata*	PPPPQPVGGGGGNRW	0.955862	Non-AMP	0.092
Uncharacterized protein OS = *Biomphalaria glabrata*	SRSPPRPF	0.904351	AMP	0.993
Uncharacterized protein OS = *Pomacea canaliculata*	HDGDGPRPCCF	0.93215	Non-AMP	0.031

**Table 5:** Selected potential bioactive peptides of the jumbo squid skin proteome predicted by *in-silico* digestions with trypsin.

Proteins	Peptides	PeptideRanker Score	Anti-Microbial Peptide (AMP)	Discriminant Score for AMP
ADP-ribosylation factor OS = *Crassostrea gigas*	CPICYDFMHTAMILPECSHTFCSFCIR	0.902646	Non-AMP	0.160
Calcium-transporting ATPase OS = *Octopus bimaculoides*	FSDDYPGFF	0.970864	Non-AMP	0.006
Calcium-transporting ATPase OS = *Crassostrea gigas*	FLQFQLTVNCVAVMVAFFGACIINDSPLK	0.979848	Non-AMP	0.281
Calcium-transporting ATPase OS=*Lottia gigantea*	FADAPFMK	0.93747	Non-AMP	0.014
Calmodulin OS = *Crassostrea gigas*	GAFFVFDR	0.915228	Non-AMP	0.003
Chitinase OS = *Todarodes pacificus*	MLAVSLLFLLAIGGVSSAGHR	0.976725	AMP	0.746
Chitotriosidase OS = *Euprymna scolopes*	MASTFATVFGVLSLCFLGLHLTNGEYK	0.984749	Non-AMP	0.106
Coatomer subunit beta’ OS = *Mizuhopecten yessoensis*	YCLCLFR	0.924855	AMP	0.579
Collagen ColAa OS = *Sepia pharaonis*	GPPGIPGLPGPK	0.93716	AMP	0.504
Collagen ColAa OS = *Sepia pharaonis*	GPPGPPGLK	0.913133	Non-AMP	0.119
Collagen ColAa OS = *Sepia pharaonis*	AGPPGFPGTPGPK	0.907398	AMP	0.682
Ficolin-2 OS = *Crassostrea gigas*	DQDNDMYVSDNCGILFPSGWWHR	0.901865	Non-AMP	0.008
Fructose-bisphosphate aldolase OS = *Mizuhopecten yessoensis*	KPWALTFSFGR	0.93422	Non-AMP	0.123
Hemocyanin OS = *Aplysia californica*	MVGYLGQALMALLLLALSNAALVR	0.993669	Non-AMP	0.380
Hemocyanin OS = *Aplysia californica*	FEPNPFFSGK	0.924588	Non-AMP	0.093
Hemocyanin OS = *Aplysia californica*	VACCLHGMPVFPHWHR	0.903581	Non-AMP	0.106
Hemocyanin OS = *Nautilus pompilius*	MATHWHSLLLFSLQLLVFTYATSDPTNIR	0.97599	Non-AMP	0.008
Hemocyanin OS = *Sepiella maindroni*	GSPIGVPYWDWTKPMK	0.917605	Non-AMP	0.027
Hemocyanin-like protein OS = *Uroteuthis edulis*	TNFFFLALIATVWLGNAETETETSK	0.90323	Non-AMP	0.062
Hemocyanin subunit 1 OS = *Euprymna scolopes*	VFVGFLLHGFGSSAYATFDICNDAGECR	0.96087	Non-AMP	0.233
Hemocyanin subunit 1 OS = *Euprymna scolopes*	LNHLPLLCLAVILTLWMSGSNTVNGNLVR	0.926117	Non-AMP	0.287
Hemocyanin subunit 1 OS = *Euprymna scolopes*	VFAGFLFMGIK	0.904542	AMP	0.865
Hemocyanin subunit 2 OS = *Euprymna scolopes*	VFAGFWFHGIK	0.943	AMP	0.506
Hemocyanin subunit 2 OS = *Sepia officinalis*	VFGGFWLHGIK	0.907156	AMP	0.739
Hemocyanin subunit 3 OS = *Sepia officinalis*	TSFLFLAFVATSWFVYAVTASK	0.905214	Non-AMP	0.136
Malate dehydrogenase OS = *Sepia officinalis*	DLFNTNASIVANLADACAQYCPK	0.965037	Non-AMP	0.251
Myosin heavy chain isoform A OS = *Octopus bimaculoides*	YQSGFIYTYSGLFCVAINPYR	0.956725	Non-AMP	0.024
Myosin heavy chain OS = *Todarodes pacificus*	NWEWWR	0.951523	Non-AMP	0.478
Myosin II heavy chain OS = *Doryteuthis pealeii*	NWQWWR	0.973264	AMP	0.959
Myosin II heavy chain OS = *Doryteuthis pealeii*	YYSGLIYTYSGLFCVVVNPYK	0.939159	Non-AMP	0.032
Neuronal acetylcholine receptor subunit non-alpha-2 OS = *Crassostrea gigas*	LLIDLCLSVLVTTLAIVSLYFYDMSDSR	0.904075	Non-AMP	0.015
Peptidyl-prolyl cis-trans isomerase OS = *Mizuhopecten yessoensis*	MAGAGIGCVLLFLLPALLSAGK	0.996478	Non-AMP	0.159
Phosphoglucomutase-1 OS = *Crassostrea gigas*	DGLWAVLAWLSVLANQNCSVEECIK	0.991266	AMP	0.904
Protein disulfide-isomerase OS = *Octopus bimaculoides*	NVFIEFYAPWCGHCK	0.907443	Non-AMP	0.053
S-syntaxin OS = *Doryteuthis pealeii*	IAILVCLVILVLVIVSTVGGVFGG	0.965343	Non-AMP	0.000
Titin OS = *Crassostrea gigas*	DGSWQNLVTVLGCLKPQFVNLQR	0.974127	AMP	0.724
Titin OS = *Crassostrea gigas*	GYPPPIISWYR	0.917986	Non-AMP	0.074
Tubulin alpha chain OS = *Octopus bimaculoides*	FVDWCPTGFK	0.923256	Non-AMP	0.010
Uncharacterized protein OS = *Arion vulgaris*	APDFIFYAPR	0.921198	Non-AMP	0.009
Uncharacterized protein OS = *Octopus bimaculoides*	FLQFQLTVNVVAVLVAFFGACTINVSI	0.978717	AMP	0.916
Uncharacterized protein OS = *Octopus bimaculoides*	YYTFFVTIFLFATTLCSTIPKPK	0.984914	Non-AMP	0.012
Uncharacterized protein OS = *Octopus bimaculoides*	LFPAFGFGAR	0.94902	AMP	0.505
Uncharacterized protein OS = *Octopus bimaculoides*	ATMLGAQGNIFFASLSCCCLILSCS	0.999233	AMP	0.879
Uncharacterized protein OS = *Octopus bimaculoides*	SGPFYIFSGGMPR	0.939205	Non-AMP	0.089
Uncharacterized protein OS = *Octopus bimaculoides*	EFSMMFR	0.931708	Non-AMP	0.001
Uncharacterized protein OS = *Octopus bimaculoides*	YGSCVPCNCNGFSNDCDPVTGECIDCQR	0.980617	Non-AMP	0.243
Uncharacterized protein OS = *Octopus bimaculoides*	HNPEGCISCFCMGVTEFCTSTSR	0.964134	Non-AMP	0.083
Uncharacterized protein OS = *Octopus bimaculoides*	APMVELCECPQGYTGVSCQECSPGYSR	0.963828	Non-AMP	0.012
Uncharacterized protein OS = *Octopus bimaculoides*	GCGCSAGQFECQNGLCINENK	0.930153	AMP	0.982
Uncharacterized protein OS = *Octopus bimaculoides*	EECMSCFCFK	0.918951	AMP	0.982
Uncharacterized protein OS = *Octopus bimaculoides*	NSEYGFACFCPQGFAGYQCDTVGER	0.906197	AMP	0.576
Uncharacterized protein OS = *Octopus bimaculoides*	MIIYILSLAGVALGVYFLSCVR	0.995663	Non-AMP	0.008
Uncharacterized protein OS = *Octopus bimaculoides*	MILTIFACLMALDIELNTSNSIQEE	0.968187	Non-AMP	0.026
Uncharacterized protein OS = *Octopus bimaculoides*	AIGALVDACGPGLCPDWADWAPK	0.948884	AMP	0.774
Uncharacterized protein OS = *Octopus bimaculoides*	QGDWTCPNPACGNNNFGWR	0.9572	Non-AMP	0.286
Uncharacterized protein OS = *Octopus bimaculoides*	GGFGGGGGGGGGMGGDR	0.928063	Non-AMP	0.065
Uncharacterized protein OS = *Octopus bimaculoides*	GFFEDDYDEYGGGYGGGMGFGGLNR	0.944869	Non-AMP	0.143
Uncharacterized protein OS = *Octopus bimaculoides*	LDDGDACLLDMGTEYCCYASDITCSYPVNGK	0.968621	Non-AMP	0.056
Uncharacterized protein OS = *Octopus bimaculoides*	MAFYTILNVVTIVLLIIVGQCR	0.998628	Non-AMP	0.031
Uncharacterized protein OS = *Octopus bimaculoides*	GGSFGFNFR	0.969779	Non-AMP	0.355
Uncharacterized protein OS = *Octopus bimaculoides*	NSTDVCNCSIYVGLFPCNECTK	0.994975	Non-AMP	0.462
Uncharacterized protein OS = *Octopus bimaculoides*	PPSPPIYFR	0.946483	Non-AMP	0.226
Uncharacterized protein OS = *Octopus bimaculoides*	CFLCATGTGTSIEVLALVTIGWCLLHATGTR	0.96344	AMP	0.768
Uncharacterized protein OS = *Octopus bimaculoides*	FDFFYK	0.96245	Non-AMP	0.032
Uncharacterized protein OS = Octopus bimaculoides	FSPIPFLFCTISGTCNFATR	0.95134	AMP	0.505
Uncharacterized protein OS = *Octopus bimaculoides*	FWELTECCPHQCLEWLSNLVTR	0.933791	Non-AMP	0.106
Uncharacterized protein OS = *Octopus bimaculoides*	DAFCSSPNFNSWLK	0.922125	Non-AMP	0.058
Uncharacterized protein OS = *Octopus bimaculoides*	NGYEEDDALIGLLNLCTAILK	0.917521	Non-AMP	0.479
Uncharacterized protein OS = *Octopus bimaculoides*	DYFWLVCR	0.911557	Non-AMP	0.001
Uncharacterized protein OS = *Biomphalaria glabrata*	QGELGDCWLLAAVASLTCNPK	0.919385	AMP	0.783
Uncharacterized protein OS = *Biomphalaria glabrata*	SPPRPFEWK	0.905581	Non-AMP	0.006
Uncharacterized protein OS = *Pomacea canaliculata*	SVFNIPPNCFSEMM	0.908085	Non-AMP	0.003
Uncharacterized protein OS = *Pomacea canaliculata*	SCLMGHGSLFGAGAGSLHLQAIAALK	0.919795	Non-AMP	0.315

## Supplementary Materials

The following are available online at https://www.mdpi.com/1660-3397/18/1/31/s1, Table S1: Peptide Spectrum Matches (PSMs), Table S2: Peptide Groups, Table S3: Proteins, Table S4: Potential bioactive peptides predicted after pepsin or trypsin digestion.
